# Diffusion Weighted Image Denoising Using Overcomplete Local PCA

**DOI:** 10.1371/journal.pone.0073021

**Published:** 2013-09-03

**Authors:** José V. Manjón, Pierrick Coupé, Luis Concha, Antonio Buades, D. Louis Collins, Montserrat Robles

**Affiliations:** 1 Instituto de Aplicaciones de las Tecnologías de la Información y de las Comunicaciones Avanzadas (ITACA), Universidad Politécnica de Valencia, Valencia, Spain; 2 Laboratoire Bordelais de Recherche en Informatique, Unité Mixte de Recherche CNRS (UMR 5800), 351, cours de la Libération F-33405 Talence cedex, France; 3 Institute of Neurobiology, National Autonomous University of Mexico, Querétaro, México; 4 CMLA, ENS Cachan, 61 av. du président Wilson 94235 Cachan, France; 5 Departament de Matemàtiques, Universitat Illes Balears, Palma, España; 6 McConnell Brain Imaging Centre, Montreal Neurological Institute, McGill University, Montreal, Canada; Beijing Normal University, China

## Abstract

Diffusion Weighted Images (DWI) normally shows a low Signal to Noise Ratio (SNR) due to the presence of noise from the measurement process that complicates and biases the estimation of quantitative diffusion parameters. In this paper, a new denoising methodology is proposed that takes into consideration the multicomponent nature of multi-directional DWI datasets such as those employed in diffusion imaging. This new filter reduces random noise in multicomponent DWI by locally shrinking less significant Principal Components using an overcomplete approach. The proposed method is compared with state-of-the-art methods using synthetic and real clinical MR images, showing improved performance in terms of denoising quality and estimation of diffusion parameters.

## Introduction

Magnetic Resonance imaging (MRI) has been intensively used to study the normal and pathological human brain. In the last decade, Diffusion Weighted Imaging (DWI) has gained a lot of attention due to its ability to measure the microscopic motion of water molecules within tissue, and hence lead to insight regarding cerebral white matter microstructure. One of the most prominent applications of DWI is Diffusion Tensor Imaging (DTI), where the directionality and the magnitude of water diffusion is estimated using a tensor model yielding images of normal and abnormal white matter fiber structure and maps of brain connectivity through fiber tracking [1] [2].

Diffusion weighted images have an inherently low signal to noise ratio (SNR) due to low signal amplitude and pronounced thermal noise, which is more evident than in conventional MRI due to extremely fast echo-planar acquisition strategies. Such low SNR makes DWI analysis complicated and biases the estimation of quantitative diffusion parameters [3]. Moreover, this limited SNR makes automated processing of these images challenging and potentially misleading.

To increase the SNR, it is a common practice to average several acquisitions in order to reduce noise variance (but without removing noise driven bias). However, this approach is time consuming in terms of acquisition and therefore not adequate for typical clinical settings where patients cannot remain still for extended periods of time.

On the other hand, denoising techniques can be applied to improve data quality as a post-processing step, thereby not increasing the scanning time. Denoising techniques applied in DWI can be divided on three categories: first, the techniques that directly filter the acquired DW images [4]–[18], second, those that regularize the tensors after estimation [19],[20] and third those that use a regularization term during the estimation/inversion [21]–[23]. However, Jones and Basser [3] demonstrated that the underestimation of diffusion anisotropy due to noise cannot be corrected once the tensor parameters are determined.

Recently, several methods that directly operate over the DWI data have reported remarkable results. For example, Wiest-Daesslé et al. [17] proposed a modification of the well-known Non-local Means method to deal with Rician noise which was further studied by Descoteaux et al. [7]. More recently, Tristan et al. [18] proposed a Linear Minimum Mean Squared Error (LMMSE) rooted approach to deal with Rician noise and the multi-component nature of DW images.

From a different point of view, Principal Component Analysis (PCA) and related approaches have been previously used for noise reduction in images [24]–[26]. In this context, noise removal can be done by a) decomposing the signal into the local principal components, then b) shrinking the less relevant components, and finally c) reconstructing back the signal. The key idea of this process is the fact that image patterns can be represented as a linear combination of a small number of basis images while the noise, being not sparse will be spread over all available components. In this sense, an interesting approach is the two steps method proposed by Zhang el al. [27] where similar patches are grouped together before PCA decomposition takes place. Alternatively, PCA can be used to learn an orthogonal basis set from the noisy data and to use it to represent noisy patches but previously zeroing small coefficients [28]. This approach takes benefit from the intrinsic sparsity properties of the images.

PCA based denoising has already been applied for MRI filtering. In Manjón et al. [29] PCA was used as a postprocessing step to remove remaining noise after the application of a multicomponent non-local means filter for multimodal MRI. Also very recently, structure adaptive and edge constrained PCA related approaches has been proposed for DWI denoising [30], [31].

In this paper, we propose a new denoising method based on local PCA designed to take into account the Rician nature of the noise present in DW images. The proposed filter takes advantage of the multi-directional nature of DW images by using local PCA decomposition to exploit the local signal profile redundancy in contrast with related PCA based methods that made use of local spatial pattern redundancy instead.

## Materials and Methods

Principal component analysis is an orthogonal linear transformation that maps the data into a new coordinate system such that the greatest variance, by any projection of the data, comes to lie on the first axes (called the first principal component), the second greatest variance on the second coordinate, and so on.

PCA-based denoising can be achieved using global information of an image series (one component per image) [26] or locally using local image patches [24],[25],[29]. The first approach, although effective, requires the number of images to be higher than the number of significant components of the image resulting is a less sparse representation. This problem can be overcome by performing PCA decomposition over small local windows instead of the whole image what significantly produces sparser representation (in the extreme case, i.e. in homogeneous areas, it can result in the sparsest representation not requiring any component to represent the data but on only the mean value of the region).

### Local PCA Denoising (LPCA)

In our proposed approach, we apply a local PCA to exploit the multi-directional redundancy of DWI patterns rather than using local spatial image pattern redundancy as done by Muresan and Parks [24]. This has the benefit of not requiring a search for similar patches within the image [27] resulting in a much faster processing.

Our method assumes that the whole directional information of DW images can be locally represented by less than the original *K* components. For this reason, the image is analyzed using a local 4D sliding block and at each position a PCA decomposition of this block is applied in order to locally find the most reduced representation of this data. The cancelation of superfluous information in the transformed data reduces noise while preserving the main features of the images.

**Figure 1:**
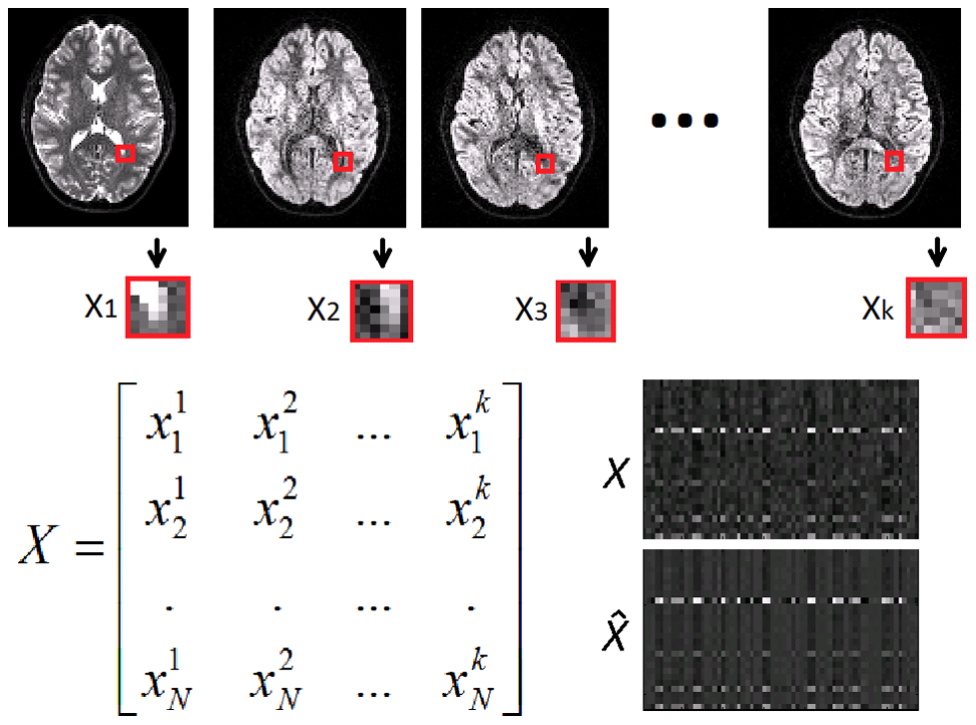
Example of local matrix X formation from a DW image series. Each block is converted into a column of matrix X. At bottom-right, an example of a real matrix X and its filtered version 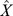 is shown to highlight the high level of profile signal redundancy present in the local matrix X.

For each point x_i_ of the image domain Ω⊂ℜ^3^, the 3D patches surrounding x_i_ in each directional image *k* are reordered as a column vector of a matrix *X* (see figure 1). *X* is thus an *NxK* matrix where *N* corresponds to the number of voxels of the 3D patch around the point of interest (N = 64 in our experiments which corresponds to 4×4×4 voxels included in the 3D patch), and *K* is the number of directional images (in DWI, *K* can range from 7 to the number of acquired directions). Therefore each row vector of this matrix represents the value of a voxel x_i_ across all *K* image directions.

PCA transforms the sample vectors into a new system where the few first components represent the most part of the variation of the original data. PCA is equivalent to compute the eigenvectors of the covariance matrix of *X, C =  X^T^ X*, where *X* has previously been normalized by subtracting to each column its own average (see [32] and [33] for a more detailed introduction to eigenvalues decomposition and PCA). The eigenvectors are stored as columns in a squared *KxK* matrix, *W*. The associated eigenvalues correspond to the amount of variation of the new components and are stored in a diagonal matrix of size *KxK, D  =  (d_kk_).* The eigenvalue decomposition of *C* writes *C = WDW^T^*. The new coordinates of the original data are computed by a simple matrix product (1).
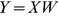
(1)


Classical thresholding algorithms cancel coefficients of low magnitude. Instead of deciding to cancel a coefficient depending on its magnitude, we take the decision based on the associated eigenvalue. By doing that, we decide to cancel the kth coefficient of the ith vector depending on the value *d_kk_.*

The use of this thresholding strategy has two advantages. First, since the new basis depends on the data the classical thresholding strategy would be less stable and second the proposed strategy is optimal for the whole set of *N* vectors. This thresholding strategy can be numerically applied by modifying the matrix *D* into 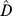. Each value in the diagonal of *D* is canceled if its magnitude is lower than a certain parameter τ. After an exhaustive search for the optimum value of τ parameter, it was set to (2.3σ)^2^, where σ^2^ is the estimated local noise variance. This threshold is set depending on σ^2^ since each element of the diagonal matrix *D, d_kk_*, actually represents the variance of the different principal components.

Finally the denoised matrix is obtained by computing the inverse PCA transformation [32], [33].
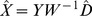
(2)where the inverse *W^–1^* is equal to the transpose of W since the matrix is orthogonal and matrix 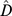 is obtained by comparing the magnitude of the diagonal with a fixed threshold as described previously. Therefore, although the final values are obtained by a matrix product, the overall process is nonlinear.

The local PCA denoising is done in an overcomplete manner since overlapping patches are used to achieve the processing of each voxel of the image volume. Due to this patch overlapping, several estimations are obtained for a given voxel since all the voxels are processed independently. Each one of these estimations is obtained by a thresholding in a different basis. The advantages of such a multipoint-wise approach compared to point-wise method are described in Katkovnik el al [34].

Therefore, for each voxel all the local estimates 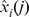 are combined from all the overlapping *j* blocks at position *i* using the following weighted average rule:
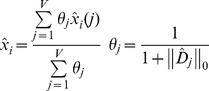
(3)where *V* is the number of overlapping blocks contributing to 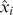 and 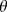 *_j_* is the weight of each block *j*, which is proportional to the inverse of the 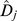 *L0* norm (i.e., the number of nonzero coefficients of the diagonal matrix 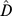 at block *j* after the thresholding operation). This approach gives more weight to estimates with more null values after thresholding. Such averaging enables to remove more noise and minimizes Gibbs artifacts [35] in a similar manner to the translation invariant denoising proposed by Coifman and Donoho [36]. The only parameter in the proposed method is the threshold value *τ*.

### Adaptation to Rician noise

Noise in magnitude MR images is usually modeled with a Rician distribution [37]. The asymmetry of the Rician distribution results in a non-constant intensity bias as it depends on SNR. Therefore, all measurements based on intensity will be biased such as Apparent Diffusion Coefficient (ADC), Fractional Anisotropy (FA), etc.

To avoid such bias, some authors have proposed to remove the bias in the squared magnitude image [17], [37]. However, this approach cannot be applied within our framework since we are not reducing the noise using the averaging principle. In our case, due to the effect of PCA thresholding, the bias in the squared domain is not constant, but dependent on intensity. However, it can be estimated theoretically and inverted in the original domain using the properties of the first moment of a Rician distribution [38].

The proposed algorithm is used to recover the mean value of the Rice distribution *R(v,σ)* with parameters *v* and *σ*, being *v* the true value we want to recover and *σ* the noise standard deviation. This expected value writes as:
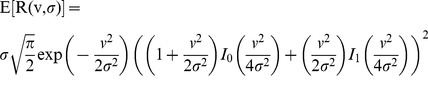
(4)where *I_0_* and *I_1_* are the modified Bessel functions of order zero and one, respectively.

Once we obtain our denoised value, we should compensate the bias by inverting previous expression and recovering the true value *v*. However, this is not possible analytically and a numerical inverse is computed. We observe that *E[R(v,σ)]/σ* can be written as a function of *φ = v/σ*.
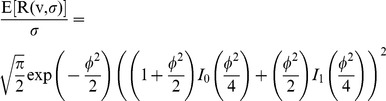
(5)


The inverse of this expression as a function of *φ* can be stored into a Look Up Table (LUT) that we denote by *η(φ)*.

The final algorithm corrects the estimated value *x* obtained by the denoising process to:
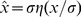
(6)


### Noise estimation in DWI

The proposed method requires the local noise level present in the images as the only input parameter. Although several methods have been proposed to estimate Rician noise level on MRI images [10],[39] few of them are able to estimate spatially varying noise patterns. Here, we propose two novel methods to provide local noise variance estimation of DW images depending on whether they have one or multiple b = 0 images.

### Multiple B = 0 image noise variance Estimator (MUBE)

If multiple non-DWI (i.e., b = 0 s/mm^2^, termed b0 henceforth) images are available in the dataset we can use image differences to robustly estimate the noise pattern. Landman et al. [40] presented a method for local noise variance estimation using the differences of two non diffusion weighted images that robustly estimated the noise pattern assuming that Rician noise can be well approximated by a Gaussian distribution if the local SNR is high enough (SNR>5).

We propose a similar approach, albeit without assuming Gaussian distributed noise by using the analytical correction scheme proposed by Koay and Basser [38].

**Figure 2:**
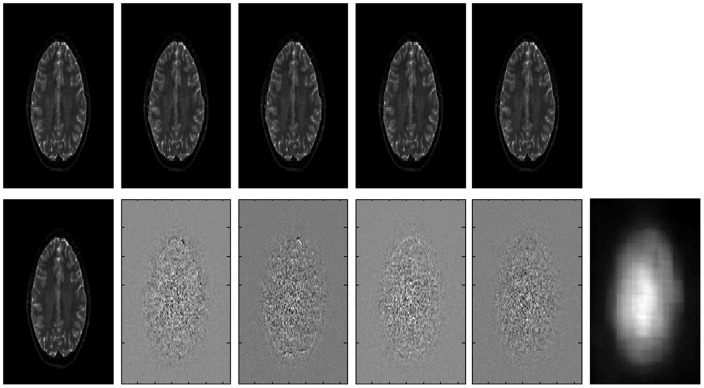
Upper row, from left to right: first to 5th b = 0 images. Lower row, from left to right: first to 5th principal components and the regularized estimated noise field. Note that the least significant component clearly shows the spatially varying noise pattern while showing no anatomy at all.

Given a set of *n* b0 images (n> = 2), we first compute global PCA decomposition of image series (i.e. we perform a PCA decomposition of a matrix formed by as many columns as image voxels and as many rows as images, see Fig. 2). In this way, first components will be associated to both signal and noise while the last components will contain mainly noise contribution. Therefore, an initial noise field estimation can be obtained just calculating the local noise standard deviation of the least significant component within regions of 3×3×3 voxels (figure 2).

However, since we are dealing with Rician and not Gaussian noise, the local standard deviation is underestimated at low SNR areas due to the asymmetric distribution of Rician noise. To correct such underestimation a correction factor was applied based on the local SNR as described by Koay and Basser [38] and used for MRI noise estimation in Coupe et al. [39].
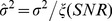
(7)where 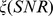 is defined as follows:



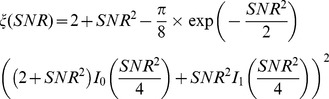
(8)

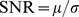
Here *µ* is the local mean, *σ^2^* is the local estimation of the noise variance and 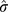 *^2^* is the corrected estimation in the Rician case.

Finally, a low-pass filter is applied to regularize the estimated noise field to provide a more regular noise field. We used a kernel size of 15 mm^3^ which was experimentally determined.

### Single B = 0 image noise variance Estimator (SIBE)

If only one non-DWI is available we need to follow a different strategy to obtain the initial noise field estimation. In this case, we can use the PCA decomposition of the gradient images instead of the b0 images (we should note here that the SNR of gradient images is significantly lower than b0 images invalidating the Gaussian assumption for all brain regions). Similarity to MUBE method, first components will be associated to both signal and noise while the last components will contain mainly noise. Thus a local noise estimation (using also a 3×3×3 voxel local regions) from this last component will enable to obtain an initial noise field estimation. Finally, this underestimated initial noise field is processed in the same manner of MUBE method to correct local noise underestimation.

## Experiments and Results

To evaluate and compare the proposed methods a set of experiments were performed with simulated and in-vivo real datasets.

### Simulated dataset

A numerical phantom consisting of a set of diffusion-weighted images was generated using the Numerical Fiber Generator (NFG) software package [41]. The simulated images were generated with a diffusion-weighted response function based on the diffusion tensor model with a fractional anisotropy of FA = 0.8, apparent diffusion coefficient ADC = 0.9×10^–3^ mm^2^/s, 7 b = 0 s/mm^2^ images and 60 DWIs along 60 uniformly distributed diffusion-gradient directions (b = 3000 s/mm^2^), with volume field of view dimensions of 100×100×100 voxels, with a voxel size of 2×2×2 mm^3^.

### In-vivo real dataset

To compare the different methods on an in-vivo real case DWI data were acquired using a 3T MR scanner (Achieva, Philips Medical Systems) equipped with a gradient system providing a maximum gradient strength of 40 mT/m and with an 8-channel phased array head coil. A standard SENSE (factor = 2) spin-echo EPI pulse sequence was used to acquire DWI data (b-value: 700 s/mm2; 21 diffusion directions) with the following parameters: TE/TR ∼60/8000 ms; FOV  = 215×215×85 mm^2^; matrix size  = 172×172 with 68 slices and a spatial resolution at 1.2×1.2×1.2 mm^3^.

### Noise estimation experiments

**Figure 3:**
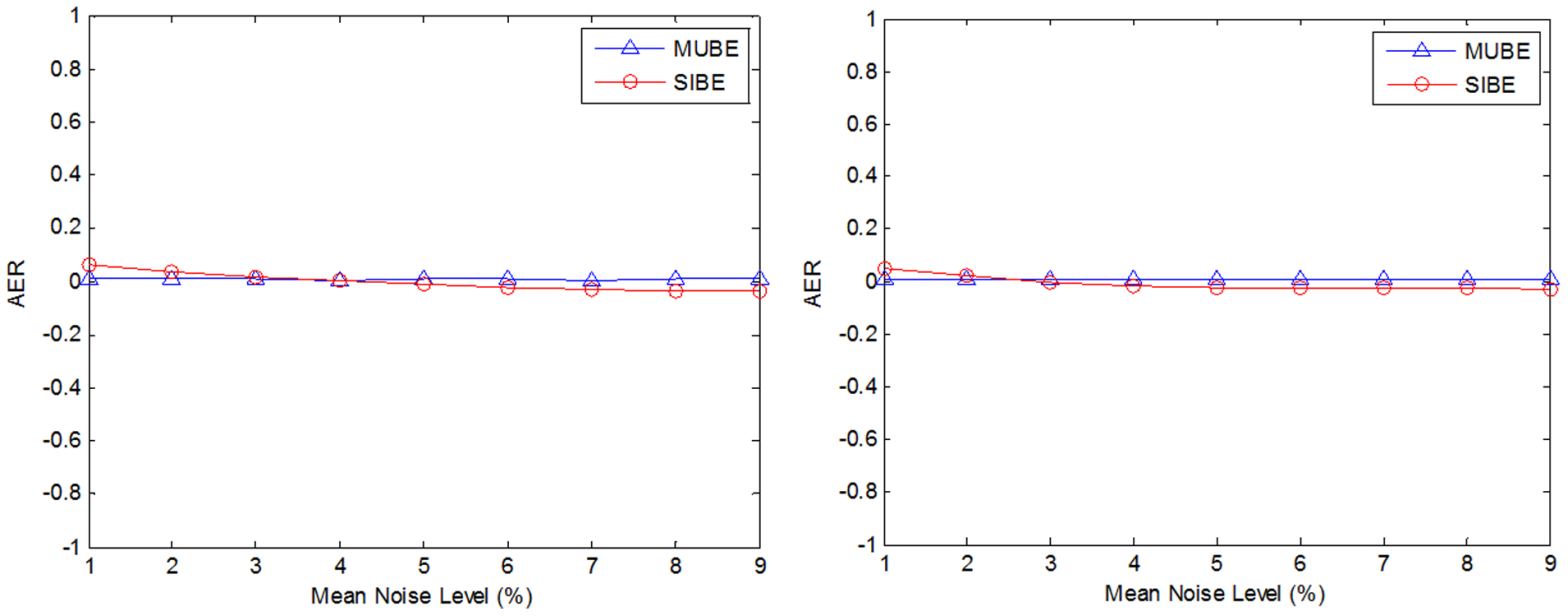
In the left figure the AER results of the two proposed methods for stationary noise is shown. At the right, the same results for spatially inhomogeneous noise is presented.

To validate the proposed noise estimation methods, an experiment consisting in estimating the level of noise after adding a known amount of Rician noise to the described synthetic phantom was performed. Both homogeneous and spatially inhomogeneous noise distributions were applied (the applied noise standard deviation ranged from 1% to 9% of the maximum b0 image amplitude). We applied inhomogeneous noise field to simulate the noise patterns related to sensitivity maps. To measure noise estimation error, the Absolute mean Error Ratio (AER) was used:
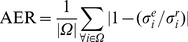
(9)where 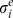 is the local estimated noise standard deviation, 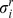 is the local real noise applied, *i* is the local coordinate and *Ω* represents the image volume. During this experiment, MUBE and SIBE methods were evaluated. The mean AER of MUBE was 0.0070, while it was 0.0276 for SIBE for homogeneous noise. The mean AER of MUBE was 0.0089 and 0.0233 for SIBE for inhomogeneous noise. As can be noticed, both methods obtained accurate results with estimation errors lower than 3% at the different noise levels (see figure 3).

### Comparison of denoising methods

Our proposed LPCA method was compared to three recently proposed methods for MRI denoising (two of them were previously used for DWI denoising):

- Joint Linear Minimum Mean Squared Error (JLMMSE) [18]. In this method all the gradient images are filtered together, exploiting the first and second order information that they share.
- Non-local Means (NLM) with Rician bias correction [17]. This method is an adaptation of Optimized blockwise NLM method [42] to deal with Rician noise. This method was applied to each DWI gradient image separately, as suggested by Wiest-Daesslé et al. [17].
- Adaptive Non-local Means (ANLM) with Rician bias correction [43]. This method is an evolution of NLM method [44] that is able to deal with spatially varying noise patterns typically present on parallel imaging. Although we have no news about the use of this method for DWI denoising we decided to include this method in the comparison since this method is able to deal with spatially varying noise fields typically present on parallel acquired DWI. This method was also applied to each DWI gradient image separately.

All three methods were run with their default parameters. In the two NLM variants, we used our own implementation of the method (Matlab mex file) while in the second case we used the command JointLMMSE utility integrated in the Slicer 3.0 software [45]. The default routines available in “dtifit” (part of the FSL [46] software distribution, FMRIB, Oxford) were used to process the data. In particular, the tensor model was fitted using least squares [47]. The tensor was diagonalized and quantitative measures extracted (i.e., FA and ADC). Probabilistic tractography was performed using *bedpostx* (also part of FSL), which uses Markov Chain Monte Carlo sampling to build distributions of diffusion parameters, including that of the principal diffusion orientation, which is then used to repeatedly seed tracks that sample from such a distribution to follow particular trajectories in a stochastic fashion. 5000 tracks were initiated per seed voxel, and all defaults defined in the *bedpostx* and *probtrackx* programs were used.

### Simulated data experiment

**Figure 4:**
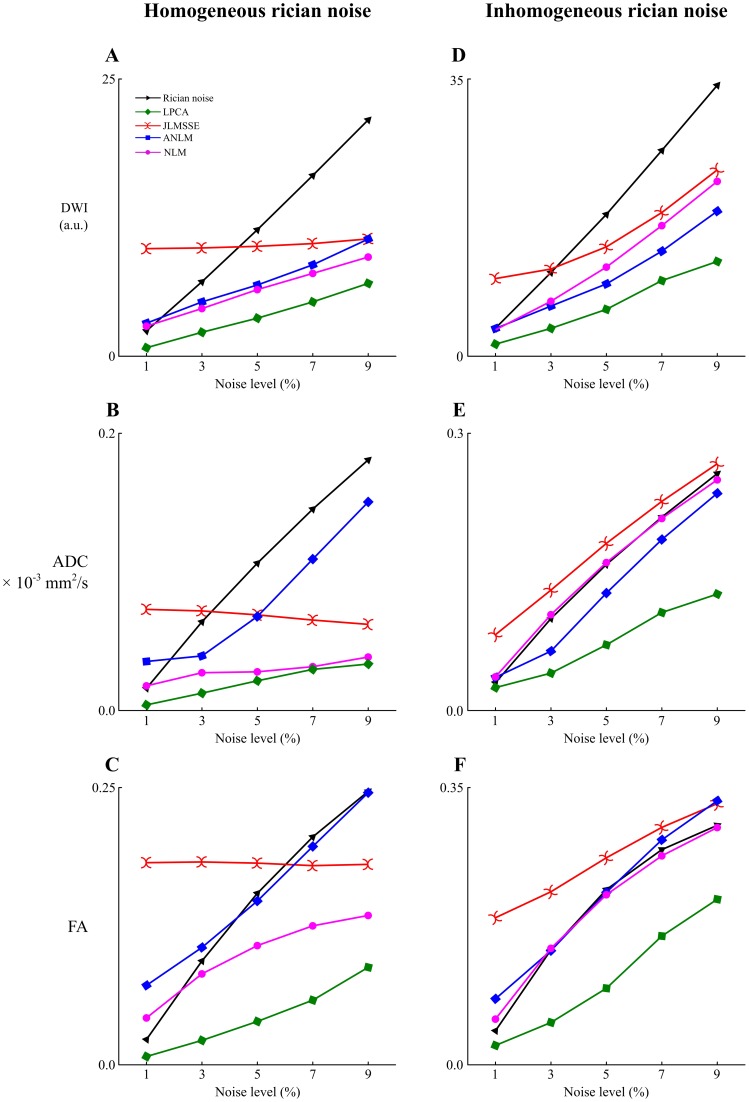
Root mean square errors obtained with the compared filters for DWI intensity (A and D), ADC (B and E) and FA (C and F). Left column: results for spatially homogeneous Rician noise. Right column: results for spatially inhomogeneous Rician noise.

The simulated phantom data was corrupted with different levels of Rician noise ranged from 1% to 9% of the maximum non-DWI signal amplitude (both stationary and spatially varying noise patterns were applied). To measure the different filter performances the root-mean-square-error (RMSE) measure was used. For the spatially stationary noise case all JLMMSE, NLM and our proposed method used the noise estimation provided by MUBE method while ANLM internally estimated the amount of noise. For the spatially varying noise case, both NLM and JLMMSE methods assume constant noise across the volume and therefore they were run using the noise level present at the background which is typically used to estimate noise level on MRI. Similarly to the stationary noise case ANLM method internally estimated the local noise level and finally our proposed method used the noise estimation provided by MUBE method. Figure 4 shows the results of the different methods for both stationary and spatially inhomogeneous noise patterns in terms of RMSE, ADC error and FA error. In all the cases the proposed LPCA method produced lower RMSE values than compared methods.

**Figure 5:**
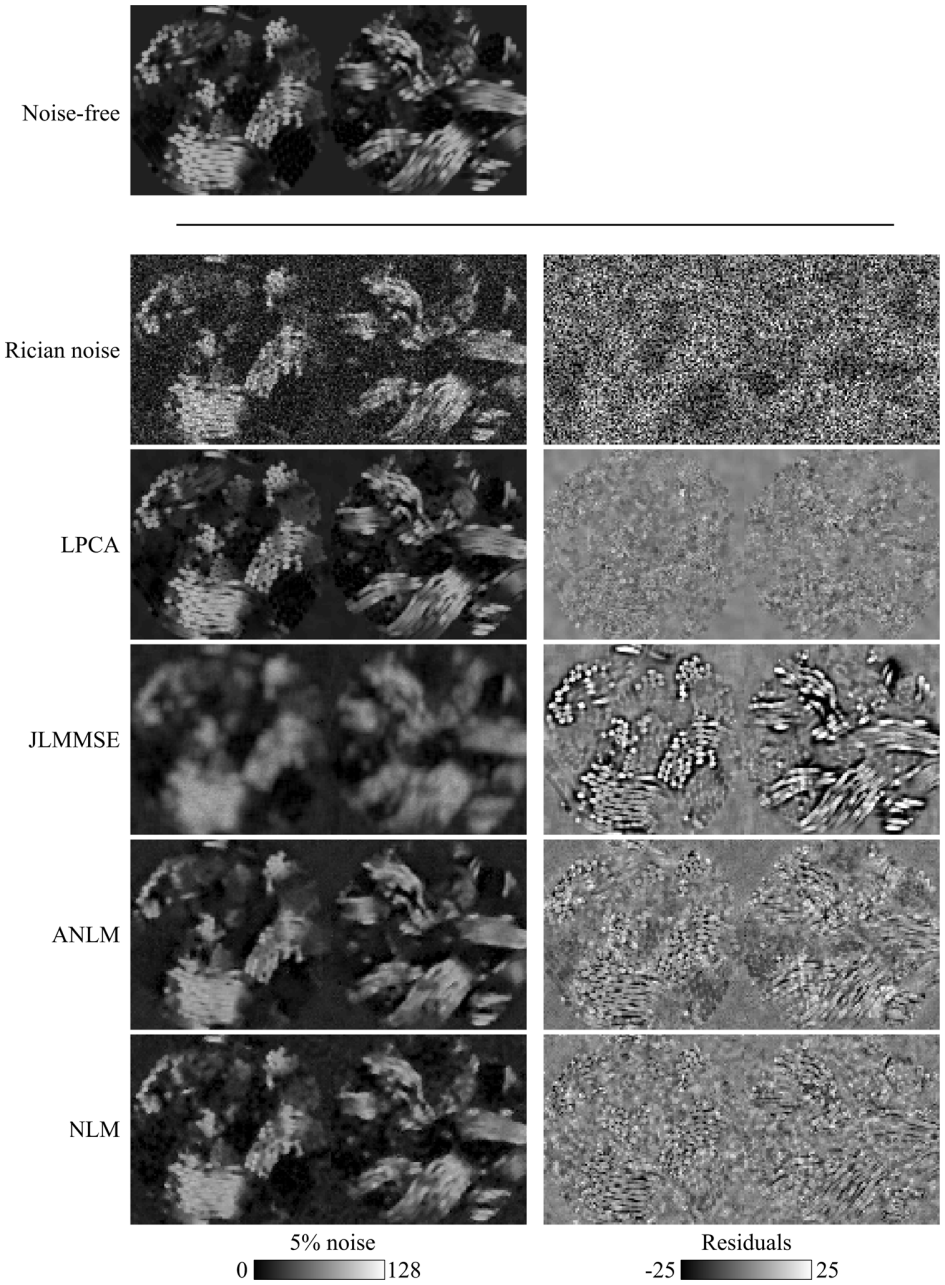
Synthetic phantom before and after denoising in the case of 5% of Rician noise. Notice the better preserved detail in LPCA-denoised images.

In Figure 5, a visual inspection of the results is proposed for the different methods compared at 5% Rician noise. The LPCA method was able to significantly reduce noise in the images while minimally affecting the original signal. Both NLM based methods slightly over-smoothed the data while the JLMMSE methods produced a more severe blurring, as it can be noticed in the image residuals (difference of the noise free and denoised images).

**Figure 6:**
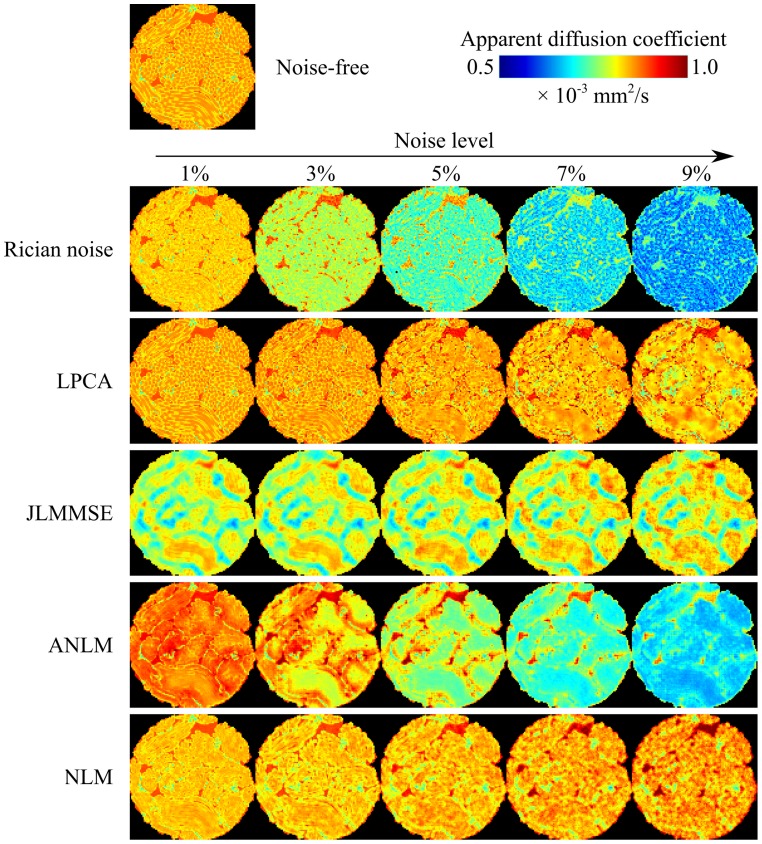
ADC results for the different methods compared. As can be noticed, the proposed method produces the less biased estimates of ADC values for all the studied noise levels.

On the other hand, the proposed method improved the estimation of the diffusion parameters (ADC and FA) for all noise levels (Figure 4) demonstrating that the LPCA method is not only able to remove high frequency noise content but also to reduce the signal bias which significantly affects the estimated parameters. Figure 6 shows the impact of Rician noise on ADC computation. We can observe that at 9% of Rician noise, the ADC values estimated from noisy phantom decreased up to 50% compared to ADC values estimated on the noise-free phantom. This demonstrates the importance of correcting for this intensity bias introduced by Rician noise. In this visual example we can see how the proposed method is able to remove noise derived bias to recover ADC values closer to ground truth compared to other filters. JLMMSE and NLM methods were able to reduce to some extent the Rician bias while ANLM method failed (probably due to an inaccurate noise estimation).

### In-vivo real data experiment

**Figure 7:**
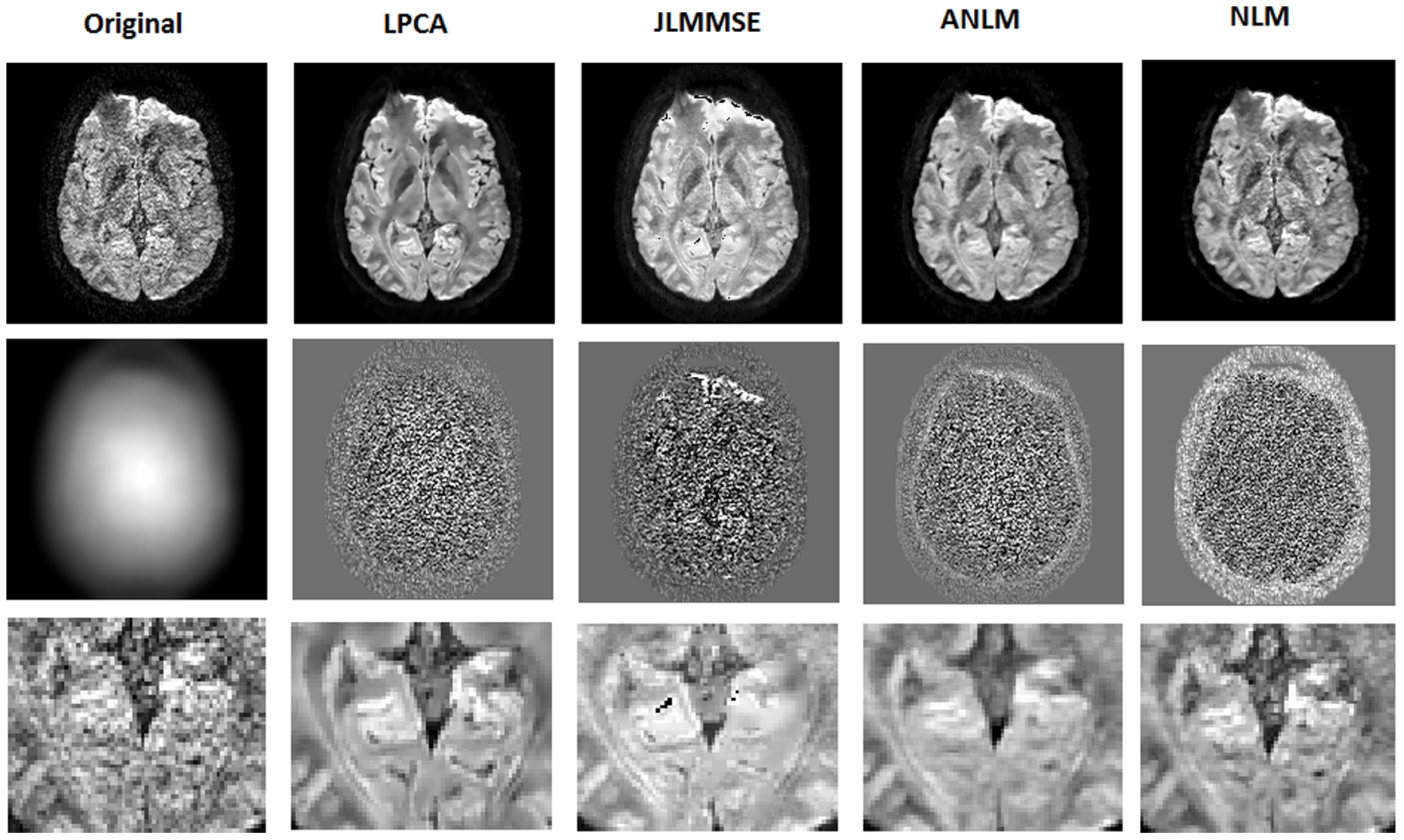
Example results on a clinical dataset. First row-Left to right: Original noisy data, LPCA results, JLMMSE results, ANLM results and NLM results. Second row-Left to right: Estimated noise field, LPCA residuals, JLMMSE residuals, ANLM residuals and NLM residuals. Third row: close-up of the results of first row.

The in-vivo real dataset was filtered with the four methods compared and the resulting images were analyzed. Noise was estimated using the proposed SIBE method since only one b0 image was available for this dataset. As expected, the noise field estimated by SIBE showed a spatially varying noise pattern due to the use of a parallel imaging SENSE sequence (see Fig. 7). ANLM, NLM and JLMMSE methods were run with their default parameters. In NLM method the noise standard deviation parameter was estimated from the image background. ANLM and JLMMSE methods internally estimated the noise level.

As can be noticed in fig. 7, the proposed LPCA method produced a clear and contrasted image. The NLM method showed some residual noise due to the noise level underestimation at image central areas. In fact, this method does not take into account the spatially varying noise. The JLMMSE method showed some artifacts at frontal areas and a slightly change of contrast probably due to an inaccurate Rician noise bias correction. ANLM perform very well but seemed to over smooth some image details.

**Figure 8:**
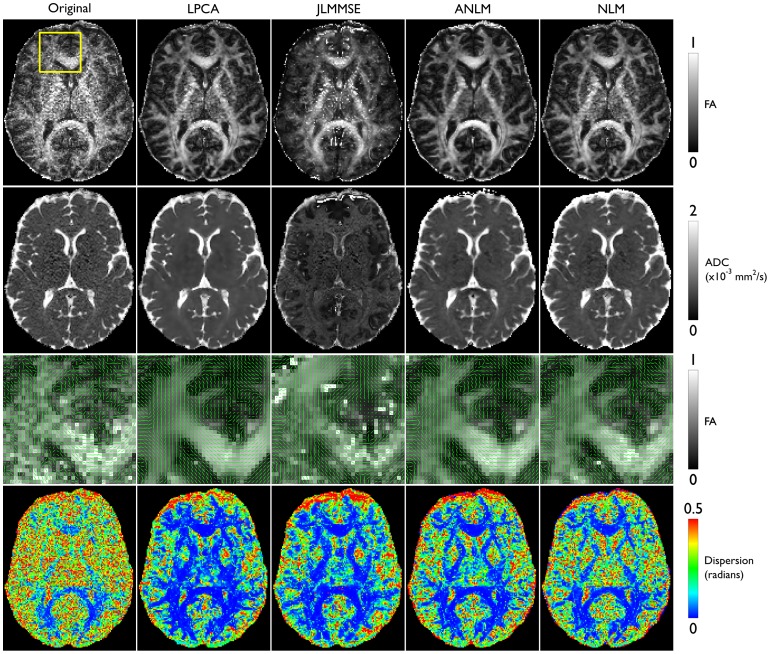
Diffusion tensor metrics. The original, low SNR, data yielded maps of Fractional Anisotropy (FA, first row) and Apparent Diffusion Coefficient (ADC, second row) of poor quality, which were improved by all denoising methods, with the exception of JLMMSE (third column). The direction of the main diffusivity, shown as green quivers (row 3), is overlaid on the corresponding FA map, showing a much smoother and coherent pattern in the LPCA-denoised data. The uncertainty of the principal diffusivity (row 4) is drastically reduced by all denoising methods, with the greatest reduction using the LPCA filter.

Diffusion measures were also performed to evaluate the performance of the four methods compared (fig. 8). It can be noted that LPCA and ANLM methods provided the greatest improvements, particularly evident in the FA and ADC maps. The deep brain structures in the FA map of the LPCA-denoised data still show slight noise, albeit considerably less than the original data. All denoising methods improved the diffusion measurements with the exception of JLMMSE (this could be caused by an inaccurate noise estimation since this method was not designed to deal with spatially varying noise patterns).It is also worth to note that the uncertainty on the estimation of the principal diffusivity was drastically reduced by all denoising methods. The greatest reduction was obtained using the LPCA method.

**Figure 9:**
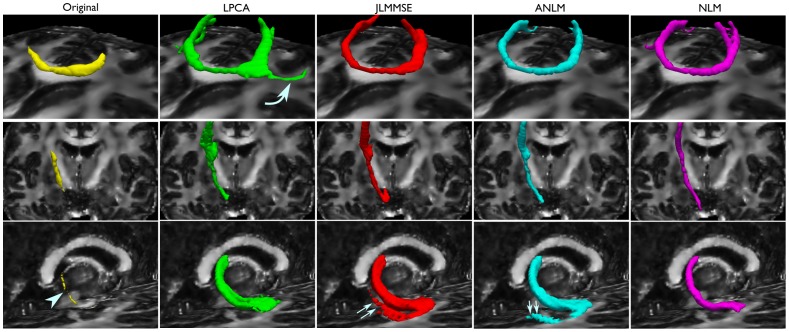
Tractography of the genu of the corpus callosum (first row), the posterior limb of the internal capsule (second row) and right crus of fornix (third row), overlaid on the FA map derived from the LPCA-denoised data. Isosurfaces represent those voxels that are intersected by at least 100 tracts in the case of the corpus callosum and internal capsule, and by at least 7 in the case of the fornix. Notice the limited propagation of all the tracts derived from the original, low SNR, data sets (first column), with the crus of the fornix barely visible (arrowhead). The corpus callosum was extended by all algorithms, but reached even farther in the LPCA-denoised data set (curved arrow). The crus of the fornix is extended and its width increased by all denoising methods, but JLMMSE and ANLM-denoised data sets produced spurious tracks (double arrows), which were not produced in the LPCA and NLM-denoised data sets.

Finally, tractography experiments were performed to evaluate the effect of the denoising algorithms on the directional information of water diffusion. Tractography of the genu of the corpus callosum, the posterior limb of the internal capsule and right crus of fornix were obtained. Tracts for the corpus callosum and internal capsule result from seeding a single voxel, whereas the fornix was seeded in 40 voxels at the level of the body of the fornix, with an AND operator at the level of the hippocampus. The same tracking criteria were used for all data sets. Figure 9 shows tractography results of the different denoised data using the compared methods. As can observed LPCA produced the most coherent results of all compared methods. JLMMSE and ANLM methods produced spurious tracks not present on LPCA and NLM-denoised data.

Regarding the processing times of the compared methods, the fastest one was the JLMMSE with only 40 seconds, the second was the proposed LPCA method with around 3 minutes and finally NLM and ANLM methods took over 8 and 15 minutes correspondently. All experiments were run on an i7 intel machine with 16 GB RAM using Matlab 2011a and Windows 7.

## Discussion

In this paper, a new method for DWI denoising is proposed and evaluated using both synthetic and real clinical data. In addition, a comparison with related state-of-the-art methods is provided. Despite its simplicity, we demonstrated improvements on DWI denoising and diffusion parameter estimation using our LPCA filter compared to previously proposed methods.

Our proposed filter removes noise in multi-directional DWI data by using an overcomplete local PCA-based decomposition. Such noise reduction capabilities can be understood due to high level of local profile redundancy which allows representing diffusion profiles using only a small number of components thus enabling to remove non signal related components (i.e. noise) efficiently. In addition, we have shown that our proposed filter not only reduces the noise present in the images, but also the bias induced by the Rician nature of the noise, thus producing diffusion parameters that better reflect the characteristics of the tissue, rather than noise-biased measurements. This has been clearly shown with the ADC and FA results where the lower parameter estimation error was coherent for all the noise levels tested. Moreover, tractography results were improved using our denoised method, considerably reducing the uncertainty of fiber directions and increasing the probability of connections between voxels within a given tract. Therefore, tracts can be reconstructed more reliably and thus the analyses of the tissue microstructure by means of tensor-derived metrics would be more sensitive.

The quantitative diffusion maps derived from the simulated data sets showed that the bias introduced by Rician noise in the estimation of quantitative diffusion parameters are minimized when denoising of the DWI is performed, with a clear advantage of the LPCA method compared to JLMME and NLM based algorithms. This was also true in the case of inhomogeneous noise, a scenario ever more commonly encountered, as parallel imaging methods are becoming commonplace. Moreover, the directional information of the diffusion data is not affected by our method, showing negligible deviation from the true principal diffusivity compared to the noise-free phantom and clearly outperforming both the noisy phantom as well as the other denoising algorithms tested. For clinical dataset, this translated into better depictions of connectivity with a higher degree of confidence.

Our current method can be easily applied to any muti-directional DWI data set, independently of the diffusion analysis performed (e.g., DTI, HARDI, q-ball, etc...), potentially improving any quantitative measure derived from them. This has immediate benefits for the analysis of brain connectivity, as well as the study of tissue microstructure in the healthy and diseased brain.
